# Goat *AKAP12*: Indel Mutation Detection, Association Analysis With Litter Size and Alternative Splicing Variant Expression

**DOI:** 10.3389/fgene.2021.648256

**Published:** 2021-05-21

**Authors:** Zihong Kang, Yangyang Bai, Xianyong Lan, Haiyu Zhao

**Affiliations:** ^1^School of Life Sciences, Lanzhou University, Lanzhou, China; ^2^College of Animal Science and Technology, Key Laboratory of Animal Genetics, Breeding and Reproduction of Shaanxi Province, Northwest A&F University, Yangling, China

**Keywords:** goat, *AKAP12* gene, insertion/deletion, litter size, mRNA expression, alternative splicing

## Abstract

A-kinase anchoring protein 12 (*AKAP12*) plays key roles in male germ cells and female ovarian granulosa cells, whereas its influence on livestock litter size remains unclear. Herein we detected the genetic variants of *AKAP12* gene and their effects on litter size as well as alternative splicing variants expression in Shaanbei white cashmere (SBWC) goats, aiming at exploring theoretical basis for goat molecular breeding. We identified two Insertion/deletions (Indels) (7- and 13-bp) within the *AKAP12* gene. Statistical analyses demonstrated that the 13-bp indel mutation in the 3′ UTR was significantly associated with litter size (*n* = 1,019), and the carriers with DD genotypes presented lower litter sizes compared with other carriers (*P* < 0.01). Bioinformatics analysis predicted that this 13-bp deletion sequence could bind to the seed region of miR-181, which has been documented to suppress porcine reproductive and respiratory syndrome virus (PRRSV) infection by targeting PRRSV receptor CD163 and affect the pig litter size. Therefore, luciferase assay for this 13-bp indel binding with miRNA-181 was performed, and the luciferase activity of pcDNA-miR-181-13bp-Deletion-allele vector was significantly lower than that of the pcDNA-miR-181-13bp-Insertion-allele vector (*P* < 0.05), suggesting the reduced binding capability with miR-181 in DD genotype. Given that alternative spliced variants and their expression considerably account for the Indel genetic effects on phenotypic traits, we therefore detected the expression of the alternative spliced variants in different tissues and identified that *AKAP12-AS2* exhibited the highest expression levels in testis tissues. Interestingly, the *AKAP12-AS2* expression levels of homozygote DD carriers were significantly lower than that of individuals with heterozygote ID, in both testis and ovarian tissues (*P* < 0.05), which is consistent with the effect of the 13-bp deletion on the reduced litter size. Taken together, our results here suggest that this 13-bp indel mutation within goat *AKAP12* might be utilized as a novel molecular marker for improving litter size in goat breeding.

## Introduction

High prolificacy in goat breeding brings huge economic values. The lambing of goats as an important reproductive trait, is directly related with the economic benefits of the goat industry, therefore the breeding of goat variety with high fecundity is essential and necessary (Wang et al., 2020a, 2021; Zhang et al., 2020). Shaanbei white cashmere goat (SBWC) is an excellent domestic goat variety with fine cashmere and meat quality. However, its potential reproductive capacity has not been fully developed and the litter size traits urgently need to be improved. Compared with the traditional Cross-breeding, molecular marker-assisted selection (MAS) has higher efficiency and accuracy (Collard and Mackill, 2008; Mills et al., 2011). By using molecular markers like Insertion/Deletion (InDel), breeders can improve the reproductive ability of goats while retaining other excellent traits, which is of great significance to the rapid development of goat industry (Li et al., 2019; Ren et al., 2019).

A-Kinase Anchoring Protein 12 (*AKAP12*) is a structurally diverse protein that shares common features to bind the protein kinase A regulatory subunit (Qasim and McConnell, 2020; Seo et al., 2021). Previous studies have suggested that *AKAP12* plays a potential role in male germ cells and ovarian granulosa cells in mammals (Erlichman et al., 1999; Binder et al., 2013). In addition, it was reported that follicle-stimulating hormone (FSH) could downregulate the expression of AKAPs in granulosa cells (Escamilla-Hernandez et al., 2008). The increased expression of *AKAP12* in the downstream FSH signaling pathway of estrogen receptor β (ERβ)-null granulosa cells suggested that FSH could only downregulate *AKAP12* under the action of ERβ, and the increased expression of *AKAP12* might lead to the isolation of PKA regulatory units, as well as an observed decrease in cAMP accumulation (Deroo et al., 2009). Together, these studies strongly suggest that *AKAP12* plays a key role in the regulation of fecundity in mammals.

Alternative splicing (AS) events can lead to multiple splicing variants of a gene and production of proteins with diverse functions therefore are necessary and fundamental in analyzing organism complexity, evolutionary pathways, and metabolic activities (Ule and Blencowe, 2019; Aísa-Marín et al., 2021). Generally, alternative splicing affects gene transcription and translation through splicing proteins and their regulatory factors, thus affecting gene functions. At present, two splicing variants of *AKAP12* gene have been documented in goats, designated as *AKAP12-AS1* and *AKAP12-AS2*. However, to date, DNA polymorphisms as well as gene expression profiles of the splicing variants of goat *AKAP12* have rarely been studied. Therefore, in this study, we investigated the genetic variants, alternative splicing and expression profiles of goat *AKAP12*, as well as their potential effects on the first-born litter size, in order to promote the application of marker-assisted selection (MAS) in goat breeding.

## Materials and Methods

### Sample and Data Collection

All experiments in this study involving animals were approved by the Faculty Animal Policy and Welfare Committee of Northwest A&F University (Ethic approval file No. NWAFAC1008). Moreover, the care and use of experimental animals completely conformed with local animal welfare laws, guidelines, and policies. We randomly collected the ear tissues of 1,019 Shaanbei white cashmere (SBWC) female goats from a local farm in Yulin, Shaanxi Province. These goats were all 2–3 years old, raised and managed under the same conditions (Cui et al., 2018; Kang et al., 2019). After weaning, all ewes were housed with a constant temperature and good ventilation, and litter sizes were recorded by the breeder. They were raised a nutritionally adequate diet and the ewes were free of disease. Goats included in the present study were randomly selected to ensure that the individuals had no genetic relationship, as much as possible (Chen et al., 2019a). The mean of litter size of SBWC goats was 1.46. In this study, we also collected the muscle, testis, brain, liver, and heart samples from six male goats, which were all healthy and at the same age. All goats used in this study were raised and managed under the same conditions after birth (Wang et al., 2020b). All tissue samples collected were fast frozen in liquid nitrogen and stored at −80°C after being brought back to the laboratory (Hui et al., 2020).

### Genomic DNA Extraction

Genomic DNA of goat ear tissue was extracted using the salting-out method. The concentration and quality of genomic DNA were detected using a Thermo NanoDrop 2000 (Thermo Fisher Scientific). The integrity of DNA and the presence of RNA and protein contamination were detected by 1% agarose gel electrophoresis (Lan et al., 2007; Hui et al., 2020; Wang et al., 2020b). Each DNA sample was diluted to 50 ng/μL and a total of 96 samples were randomly selected to construct DNA pools. Touchdown PCR was used for identification of genetic variations as well as individual genotyping (Chen et al., 2019b).

### RNA Extraction and Quantitative RT-PCR

The total RNA from the tissue samples was extracted by using the Trizol (TaKaRa Biotech Co. Ltd., Dalian, China) method, which mainly includes steps like sample cracking, extraction, precipitation, and dissolution. The same amount of RNA of each sample was reverse transcribed according to the instructions of the reverse transcription kit (TaKaRa Biotech Co. Ltd.). Primers for quantitative RT-PCR analysis are shown in Table 1, and the mRNA expression was detected using the CFX96 Real-Time PCR Detection System (Bio-Rad, United States) as described previously (Jia et al., 2015). The fluorescence signal was detected at the end of each cycle, and three replicates were set for each sample.

**TABLE 1:** Primers for PCR amplifications.

Primer names	Sequences (5′–3′)	Fragment (bp)	Function	Location
9-bp	F: GGGTCACTGCTTTACATCCGTT	122/122	Indel detection	3′ UTR/3′ UTR
	R: ATGGTGGCATTGTTTCAGTACCT			
7-bp	F: TGTTTAATGGCGGTAGA	149/156	Indel detection	Intron 3/Promoter
	R: GAGGGTTTCAGAGTTGC			
13-bp	F: CACTCATCCTACTGGCAT	179/192	Indel detection	3′ UTR/3′ UTR
	R: TGTTAATAGCGTTCCTCC			
qPCR-AS1	F: AGAAGCCTTGCCTCAGGAGTTTG	156	qPCR	Exon 1–2
	R: CGCCTTCTCCAAATCGCAGG			
qPCR-AS2	F: TCCTCACGATCACAGTTGGAC	110	qPCR	Exon 1–2
	R: TCCCTCCTTCGTGATGTCCT			
GAPDH	F: AAAGTGGACATCGTTGCCAT	116	Internal control	Exon 3–4
	R: CCGTTCTCTGCCTTGACTGT			
pcDNA3.1-miR-181	F: GG**GGTACC**CCTCCAGATCCTCGCAGAT	337	Overexpression	
	R: CC**CTCGAG**GATCACGCTCGCACAATGC			
psi-CHECK2	F: CCG**CTCGAG**CACTCATCCTACTGGCAT	179/192	Targeting detection	3′ UTR
	R: AAGGAAAAAA**GCGGCCGC**TGTTAATAGCGT TCCTCC			

**The bold font refers to the sequence of the restriction enzyme cutting site, the underlined font refers to the protective bases.**

### Plasmid Construction

We used RNAhybrid^1^ to predict the potential miRNA binding sites of the goat *AKAP12* 13-bp region. The 179/192-bp DNA sequence containing the 13-bp region was cloned into psiCHECK-2 using T4 DNA Ligase and the insert was released by *Xho*I and *Not*I digestion (TaKaRa Biotech Co., Ltd.). The miR-181 sequence was cloned into the pcDNA3.1 vector (Invitrogen) as described previously (Kang et al., 2020; Hao et al., 2021), and the insert was released by *Kpn*I and *Xho*I digestion.

### Cell Culture and Luciferase Reporter Assays

Human embryonic kidney 293T (HEK293T) cells were seeded in a 60-mm cell culture dish, and 4 mL of the growth medium was added. The details of the cell culture condition have been described previously (Chen et al., 2018). Before transfection, trypsin was used to digest the cells from the culture dish, and after centrifugation, 3 × 10^4^ cells were seeded into 96-well plates and cultured overnight. After transfection for 24 h, the medium was discarded, 50 μL of 1 × PLB was added to each well, and the cells were shaken at room temperature for 30 min until full lysed. Cell lysates (5 μL) were added to the 96-well ELISA plate, and luciferase activity was measured using 20 μL of LARII and Stop&Go (Promega, Heidelberg, Germany). The data were processed and calculated as: luciferase activity = Renilla luciferase/firefly luciferase.

### Statistical Analyses

Allele, genotype frequencies, and linkage disequilibrium (LD) analyses were conducted using the SHEsis software^2^. Genetic parameters were calculated using the Nei’s methods (Table 2; Nei and Roychoudhury, 1974). According to the correlation coefficients (D′/r^2^), the pattern of pairwise LD between the indel loci was estimated and visualized. The case of *D*′ = 1 or *r*^2^ = 1 is known as complete LD. Values of *D*′ < 1; *r*^2^ > 0.33 indicate strong LD. A least-squares mean (LSM) test was used to determine the litter size of goats with different indel genotypes according to the formula Y_ij_ = μ + HYS_i_ + G_j_ + e_ij_, where Y_ij_ is the phenotypic value of litter size, μ is the overall population mean, HYS_i_ is the fixed effect of the herd-year-season, G_j_ is the fixed effect of genotype, and e_ij_ is the random error (Cui et al., 2018).

**TABLE 2:** Genotype and allele frequency of two indel loci within *AKAP12* gene in Shaanbei white cashmere (SBWC) goats.

Loci	Rs number	Observed genotypes (Sample size)	Frequencies		*Ho*	*He*	PIC	HWE χ^2^ (*p*-values)
			Genotypes	Alleles				
7-bp (n = 665)	rs636798034	II (14)	0.021	0.212 (I)	0.666	0.334	0.278	13.613 (*P* = 0.0002)
		ID (254)	0.382	0.788 (D)				
		DD (397)	0.597					
13-bp (*n* = 1019)	rs639087618	II (12)	0.012	0.122 (I)	0.786	0.214	0.191	0.819 (*P* = 0.365)
		ID (224)	0.220	0.878 (D)				
		DD (783)	0.768					

**Ho, homozygosity; He, heterozygosity; PIC, polymorphism information content; HWE, Hardywenberg epuilibrium.**

For gene expression analysis, on the premise of ensuring amplification efficiency, *GAPDH* was used as the internal reference gene to correct the mRNA expression level, and 2^−ΔΔCt^ method was used to calculate the relative mRNA expression levels (Livak and Schmittgen, 2001).

## Results

### Identification of Novel *AKAP12* Indel Loci

**FIGURE 1:**
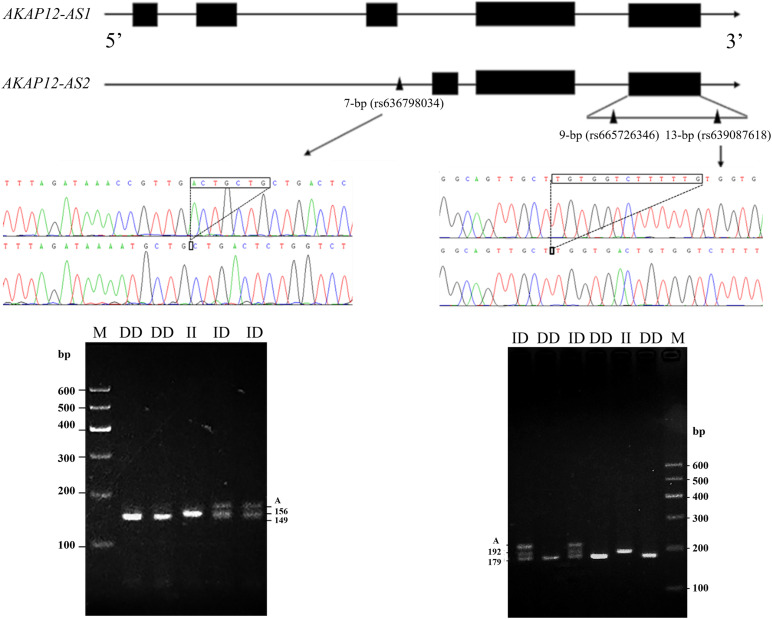
The electrophoresis diagrams and sequencing diagrams of goat *AKAP12* gene indel loci. Black boxes: exon; Black triangle: mutation site; “A” means heteroduplex.

This study referred to three indel loci that were documented in the NCBI database^3^, and their RS numbers were rs665726346 (9-bp), rs636798034 (7-bp), and rs639087618 (13-bp), respectively. The two indel loci (rs636798034 and rs639087618) were confirmed to be exist in this population. A DNA mixing pool was used as a template for PCR amplification. According to the results of gel electrophoresis, a 7-bp indel in the promoter of *AKAP12-AS2* (NC_030816: g.83323del ACTGCTG) and a 13-bp indel in the 3′ UTR (NC_ 030816.1: g.110266del TGGTCTTTTTGTG) were identified by PCR amplifications (Figure 1) using primers as shown in Table 1.

### Genotype, Allele Frequency, and Linkage Disequilibrium Analysis

**FIGURE 2:**
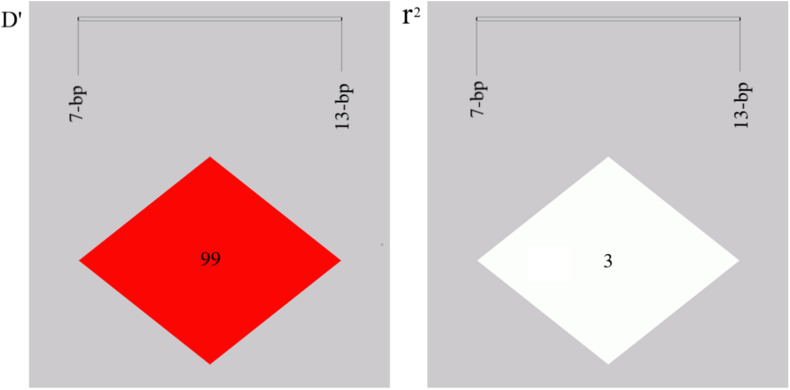
Linkage disequilibrium plot of the *AKAP12* gene two indel loci.

Both indel loci contain three genotypes: homozygotic deletion type (DD), homozygotic insertion type (II), and heterozygote type (ID). The allele and genotype frequencies are shown in Table 2. The PIC values indicated that the 7-bp indel locus had moderate polymorphism (PIC = 0.278), while the 13-bp indel locus had low polymorphism (PIC = 0.191) in SBWC. The genotypic frequency of the 13-bp indel locus was in accordance with the Hardy-Weinberg equilibrium (HWE) (χ^2^-test, *P* > 0.05) while the 7-bp indel locus was not conform to HWE (*P* < 0.05) in this investigated goat population. Then, the LD of the indel loci was analyzed and the result showed that the r^2^ value was low (0.03), while D′ value was high (0.99) (Figure 2), suggesting the minimal historical recombination between 7- and 13-bp indel loci.

### Association Analysis Between First-Born Litter Size and Indel Genotypes

Next, the associations between *AKAP12* indel loci and their productive performance in female goats (single kid and multiple kids) were investigated. The results of the litter size relevance analysis are shown in Table 3. Since no significant association was identified between the genotypes of the 7-bp indel locus and the litter size in 665 goats, we did not expand the sample size further in this locus. Interestingly, the 13-bp indel locus was significantly associated with the first-born litter size: the individuals with homozygote DD had a smaller first-born litter size, in comparison with the individuals with II and ID genotypes. We further analyzed the distribution of the genotype with the two indel variations in single and multi-lambing goat populations using the chi-square test. Consistent with the above correlation analysis, no significant difference was observed in the 7-bp indel genotype distribution, while the 13-bp indel demonstrated a significant differential genotype distribution (*P* < 0.01; Table 4).

**TABLE 3:** Associations of two indel loci with first-born litter size in Shaanbei white cashmere (SBWC) goats.

Loci	Rs number	Genotypes			*p*-values
		II	ID	DD	
7-bp	rs636798034	1.36 ± 0.15 (*n* = 11)	1.51 ± 0.04 (*n* = 216)	1.53 ± 0.03 (*n* = 313)	0.635
13-bp	rs639087618	1.70^AB^ ± 0.15 (*n* = 10)	1.64^A^ ± 0.04 (*n* = 192)	1.45^B^ ± 0.02 (*n* = 631)	0.000078

**Columns with different letters (A, B) mean P < 0.01.**

**TABLE 4:** Genotype distribution between goats with single- and multi-lamb in Shaanbei white cashmere (SBWC) goats.

Loci	Rs number	Types	Sample size	Genotypes			Genotype frequencies			Independent χ^2^-value, df, *P*-value
				II	ID	DD	II	ID	DD	
7-bp	rs636798034	Single compatriot	278	7	112	159	0.025	0.403	0.572	χ^2^ = 0.721 df = 2 *P* = 0.697
		multi- compatriots (≥2)	262	4	104	154	0.015	0.397	0.588	
13- bp	rs639087618	Single compatriot	432	3	73	356	0.007	0.169	0.824	χ^2^ = 22.501 df = 2 *P* = 0.000013
		Multi- compatriots (≥2)	398	7	119	272	0.018	0.299	0.683	

### The 13-bp Variation Influenced the Binding of miR-181 to Goat *AKAP12* 3′ UTR

**FIGURE 3:**
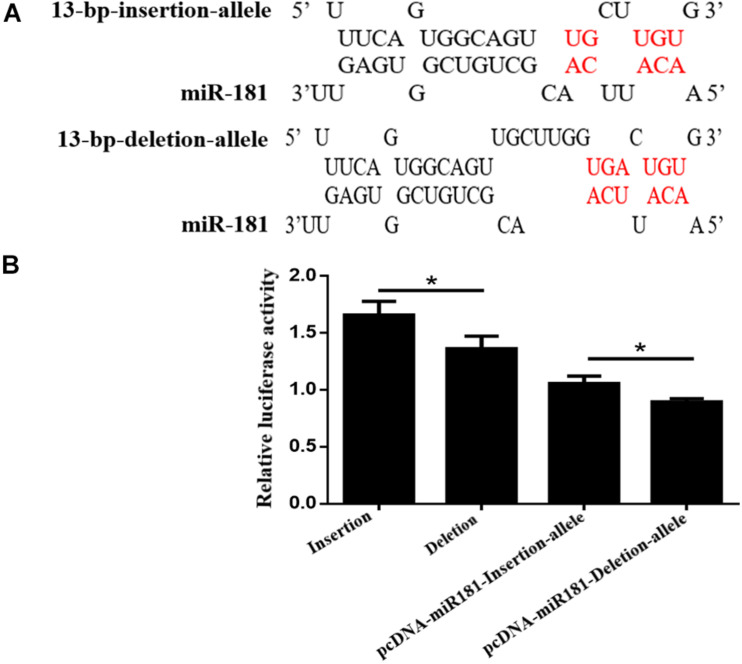
**(A)** The 13-bp indel locus influences the binding of miR-181 and goat *AKAP12* 3′ UTR. **(B)** HEK293T cells transfected with the psiCHECK-2 plasmids (DD or II types) or co-transfected with pcDNA-miR-181. The relative luciferase activity was plotted on the Y axis and data were presented as means ± SE. *P < 0.05.

Bioinformatic analysis using RNA hybrids predicted that the 13-bp deletion sequences within the *AKAP13* gene could bind to the seed region of miR-181 which was reported to suppress porcine reproductive and respiratory syndrome virus (PRRSV) infection by targeting PRRSV receptor CD163 and affect litter size. Therefore, luciferase activity evaluation for 13-bp indel in 3′ UTR biding with miRNA-181 was performed. Our results showed the binding of miR-181 and goat *AKAP12* 3′ UTR bases decreased when the 13-bp deletion was present (Figure 3A). In order to verify this predicted mechanism, the psiCHECK-2 plasmids (DD or II types) were transfected or co-transfected with pcDNA-miR-181 into the HEK293T cells, and the results indicated that the luciferase activity of II genotype vector was significantly higher than that of the DD genotype vector, and the luciferase activity of pcDNA-miR-181-Insertion allele vector was significantly higher than that of pcDNA-miR-181-Deletion allele vector (Figure 3B).

### Identification of Genetic Variants Regulating *AKAP12* Expression

**FIGURE 4:**
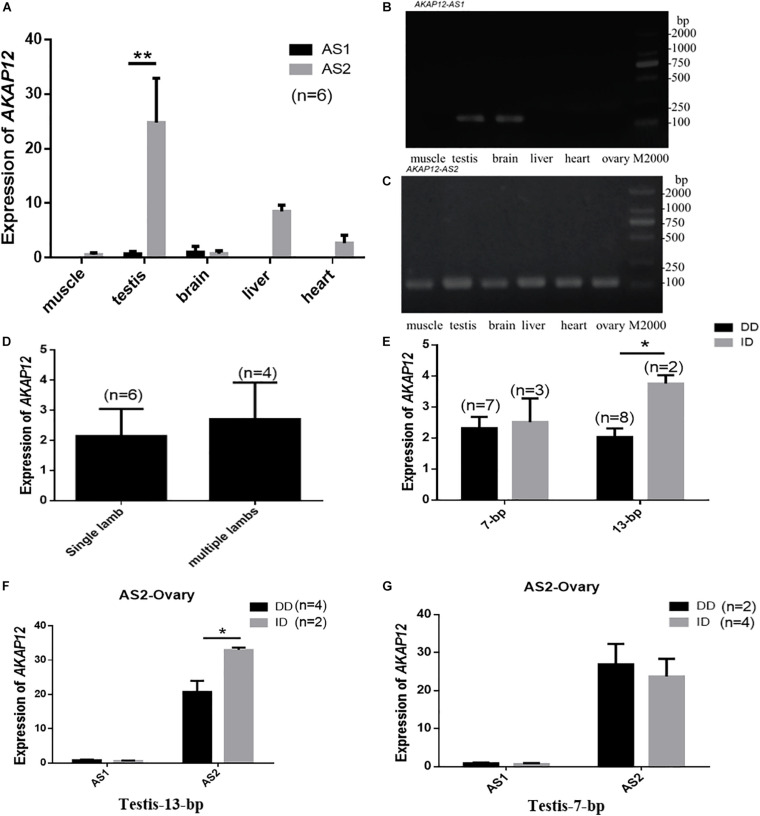
*AKAP12* mRNA expression patterns in Shaanbei white cashmere goat. **(A)** Differential expression of *AKAP12-AS1* and *AKAP12-AS2* in different tissues of male goats. **(B)** RT-PCR analysis of *AKAP12-AS1* in different tissues. **(C)** RT-PCR analysis of *AKAP12-AS2* in different tissues. **(D)** Differential expression of *AKAP12* gene in ovary tissue of goats with single-lamb or multi-lambs. **(E–G)** The correlation between *AKAP12* mRNA expression in the ovary and testis and different genotypes of the 7-bp and 13-bp indel locus. Data represent means ± SE. *P < 0.05, **P < 0.01.

Given that mRNA expression and spliced variants may also account for Indel genotypic effects on phenotypic characteristics, we detected the mRNA expression of the spliced variants in different tissues of male goats and ovaries of female goats. Two alternative *AKAP12* splicing, named *AKAP12-AS1* and *AKAP12-AS2* and their sequence alignment analysis were shown in the file S1. Our results revealed that *AKAP12-AS2* was expressed in all tissues, whereas *AKAP12-AS1* was only expressed in the testis and brain. Notably, *AKAP12-AS2* exhibited the highest mRNA expression levels in the testis tissue (Figures 4A–C). Interestingly, the ovary *AKAP12-AS2* expression in goats with multiple lambs is relatively higher than those with single lamb (Figure 4D).

In addition, the genotypes of 7-bp indel locus didn’t influence the *AKAP12* mRNA expression in both testis and ovary tissues (*P* > 0.05) (Figures 4E,G), whereas for the *AKAP12-AS2* 13-bp indel locus, the expression level of homozygote DD was significantly lower than that of individuals with heterozygote ID, in both testis and ovarian tissues (*P* < 0.05) (Figures 4E,F), which consistently explained the negative effects of the 13-bp deletion on the reduced goat litter size.

## Discussion

Reproductive capacity of goats are quantitative traits controlled by multiple genes with low heritability, therefore it is difficult to achieve significant improvements with traditional breeding. Compared with the classical cross-breeding, molecular marker-assisted selection (MAS) has remarkably higher efficiency and accuracy (Collard and Mackill, 2008; Mills et al., 2011). Therefore, we can improve the reproductive ability of goats while retaining other excellent economical traits by using molecular markers. *AKAP12* has a common function of binding to the regulatory subunit of protein kinase A and protein kinase C signaling in regulation (Su et al., 2010). Previous studies have verified that the *AKAP12* gene plays potential roles in male germ cells and ovarian granulosa cells (Erlichman et al., 1999; Binder et al., 2013). However, to the best of our knowledge, there has been no relevant report on the effects of *AKAP12* polymorphisms on the reproductive performances of goats.

In this study, we identified two novel indel loci (7- and 13- bp) within *AKAP12* gene by PCR amplifications. The frequency of “D” allele was greater than that of “I” allele for both loci, and the minimum allele frequencies of 7 and 13-bp were 0.212 and 0.122, respectively. In addition, the genotypic frequency of the 13-bp indel locus was in accordance with the Hardweinberg equilibrium state (*P* > 0.05), while the 7-bp indel locus was not (*P* < 0.05), which might result from the effective artificial selection during breeding (Zhao et al., 2013).

For the association analysis, in one way, practically we analyzed the effect of indel loci on first-born litter size of goats via two steps. Initially 665 individuals were used to analyze the correlation between first-born litter size and two indel mutations and the results showed that there was no significant association between the 7-bp indel locus and litter size, while the 13-bp locus was significantly correlated with goat litter size in the same population. Thus, we further extended the sample size from 665 to 1,019 and verified the association of the 13-bp indel locus with litter size. The individuals with DD genotype had fewer first-born litter sizes than those with the II and ID genotypes, thereby indicating that the allele “I” of *AKAP12* has a positive effect on the fertility of SBWC. Furthermore, linkage disequilibrium (LD) analyis was performed between 7 and 13-bp loci, and the low r^2^ value indicated that there was no historical recombination between these two loci, which partially explained their different genetic effects on goat litter size.

In another way, we further analyzed the distribution of genotypes of the two indel loci in single and multi-lambing goat populations using the chi-square test. In different litter size types, there was not significant difference in the 7-bp indel genotype distribution, while in the 13-bp indel locus, significant difference in the genotype distribution was observed. In combination with the linkage analysis, our results demonstrate that the 7 and 13-bp indel loci do not affect litter size traits synergistically.

Based on the above statistical analyses, we revealed that the 13-bp locus significantly affected goat litter size in a good sample size. Herein, we gave the following explanations:

Firstly, as the 13-bp indel mutation was located on the 3′ UTR of goat *AKAP12*, we hypothesized that this indel region might be the binding site of specific miRNAs thereby influencing the *AKAP12* gene expression. It is well-known that miRNAs can bind to the 3′ UTR of target mRNA to inhibit gene expression (Abdollahzadeh et al., 2019). Thus, further bioinformatics analysis demonstrated that the 13-bp deletion sequences within the *AKAP13* gene could bind to the seed region within the miR-181 which has been reported to suppress porcine reproductive and respiratory syndrome virus (PRRSV) infection by targeting PRRSV receptor CD163 and affect reproductive capacity (Gao et al., 2013; Guo et al., 2013). In addition, it has been well established that genes related to embryo implantation and placental formation were regulated by miR-181a and miR-181c (Su et al., 2014). Therefore, luciferase activity evaluation for 13-bp indel in 3′ UTR biding with miRNA-181 was performed. As expected, the luciferase activity of pcDNA-miR-181-13bp-Deletion-allele vector was significantly lower than that of the pcDNA-miR-181-13bp-Insertion-allele vector, suggesting that the locus with DD genotype exhibits significantly reduced binding capability with miR-181, partially accounting for the above associative analysis. Thus, the 13-bp indel might affect litter size traits of goats by interfering the binding of miR-181.

Moreover, genetic variations can affect gene expression thereby affecting phenotypic characters. Thus, we speculated that the results of the associative analysis might be caused by the influence of the genotypes of the indel locus on the expression levels of *AKAP12* mRNA. Therefore, we detected the mRNA expression of the spliced variants in different tissues of male goats and ovaries of female goats. The results showed that *AKAP12-AS2* was expressed in all tissues, but *AKAP12-AS1* was only expressed in the testis and brain. Notably, *AKAP12-AS2* exhibited the highest expression levels in testis tissues. Importantly, for the *AKAP12-AS2* 13-bp indel locus, the expression levels of homozygote DD were significantly lower than that of individuals with heterozygote ID, in both testis and ovarian tissues, which is consistent with the negative effects of the 13-bp deletion on the reduced litter size. It has been well established that alternative splicing might affect gene expression and translation through splicing proteins and other regulatory factors, thus affecting gene functions (Ule and Blencowe, 2019; Aísa-Marín et al., 2021). Thus, it’s tempting to speculate that the 13-bp indel variation affects the binding of miRNAs, which in turn affected gene expression, resulting in a phenotypic difference.

Briefly, our study here revealed that the 13-bp indel within goat *AKAP12* gene significantly affected the first-born litter size by interfering with the binding of miR-181 and the expression of *AKAP12* spliced variants, therefore might promote the application of MAS in goat breeding.

## Data Availability Statement

The datasets presented in this study can be found in online repositories. The names of the repository/repositories and accession number(s) can be found below: https://www.ncbi.nlm.nih.gov/genbank/, rs636798034 and https://www.ncbi.nlm.nih.gov/genbank/, rs639087618.

## Ethics Statement

The animal study was reviewed and approved by the Faculty Animal Policy and Welfare Committee of Northwest A&F University (Ethic approval file No. NWAFAC1008).

## Author Contributions

ZK and YB performed the experiments, analyzed the data, and prepared the manuscript. XL and HZ designed the experiments, discussed the results, and revised the manuscript. All authors contributed and approved the current submission.

## Conflict of Interest

The authors declare that the research was conducted in the absence of any commercial or financial relationships that could be construed as a potential conflict of interest.

## Footnotes

1 https://bibiserv.cebitec.uni-bielefeld.de/rnahybrid/

2 http://analysis.bio-x.cn

3 https://www.ncbi.nlm.nih.gov/

## Supplementary Material

Supplementary File 1 Sequence alignment of *AKAP12-AS1* and *AKAP12-AS2*.

## Abbreviations

bp: base pairs
*AKAP12*: A-kinase anchoring protein 12
indel: insertion/deletion
II: insertion/insertion
ID: insertion/deletion
DD: deletion/deletion
MAS: marker-assisted selection
SNPs: single nucleotide polymorphisms
SVs: structural variants
PCR: polymerase chain reaction
PKA: protein kinase A
PKC: protein kinase C
FSH: follicle-stimulating hormone
Ho: homozygosity
He: heterozygosity
PIC: polymorphism information content
HWE: Hardy-Weinberg equilibrium
AS: alternative splicing
qPCR: quantitative polymerase chain reaction.
