# Supplementary material for: Goat AKAP12: Indel Mutation Detection, Association Analysis With Litter Size and Alternative Splicing Variant Expression
Source: Front Genet. 2021 May 21;12:648256. doi: 10.3389/fgene.2021.648256 (PMC8176285; doi:10.3389/fgene.2021.648256)
Supplement: Supplementary File 1 — Sequence alignment of AKAP12-AS1 and AKAP12-AS2. [file Data_Sheet_1.PDF]

```
                *        20         *        40         *        60         *        80         *       100
AS1 : ATGTTCAGTGGCAAGCAGTCTGAAGGAAGGAAAACGGGGATCTAGGTTCGCATCTGGGCGAGTTGAGAGTGGGCTGCAGCAGAAGCCTTGCCTCAGGAGTTT :  102
AS2 : ------------------------------------------------------------------------------------------------------ :    -

              *       120         *       140         *       160         *       180         *       200
AS1 : GCCGCGAGCGCGTCTCCTTCATTCGCAGTCTGGGCGGGTTTGGAGGAGCACGGCGGGAGACTGAGGCGCTCTTGGGACTTTGCGAGTGCACGGCTTCTGGCT :  204
AS2 : ------------------------------------------------------------------------------------------------------ :    -

            *       220         *       240         *       260         *       280         *       300
AS1 : CTTCTCCGCGTCCCTGCGATTTGGAGAAGGCGTTCACCAAGCGGCTAGCCACCGGAGAACCGCAGACGAGCCATGGCGCCGGAGCTCCACCGAGCAGCGG :  306
AS2 : -----------------------------------------------------GGTGGTGGAGGGCTGGTGGGTCCTCGTATAATTAGCCAGGGAAAGGC :   49
                                                             GG G    G  G C  G     G  C C    A  T   C G G A  G

          *       320         *       340         *       360         *       380         *       400
AS1 : AGCCCGGAGCAGCCGGAGGCGGGAGCGCGACGCCGCCGAGCCCGAGCCAGCGGCGGCGGCTCTGCGGCGGAGGCGGCGCCCGGCACGCCGGGAGACCCG :  408
AS2 : AGCCTCAGTCCGCTTCTGGCTGCAGCTCCGACGGCACCTTTGGTTTTCCTGATC-------TTCCGAGGTGGCCTGGCGCTCAGCTCTTTGCTGATCCCA :  144
      AGCC    C GC    GGC G     CGACG C  C   G     CC             TC G GG  G    GGCGC C GC C   G      CCC

        *       420         *       440         *       460         *       480         *       500         *
AS1 : GCCATCGCCCC--TACGGACCCCGCCACCAAGCTCCTGCAGAAGAATGGCCAGCTGTCCACCGTCAACAGCTTAGCTGAGCAAGGCGAACTCGGCCTCCAGG :  508
AS2 : GCGTAGGCTGCGGTGGGGAGCCGCTGACAAGACCCCTGCGGCTCCGGCCACCCGCCC-----TAGGAGGT---CGAGGCTCGCCGCTCTCTCCGCTCA-- :  236
      GC    GC  C  T  GG   CCGC   CAAG  CC           GGCCA C G CC      A  AG T   C   GC  G CG  CTC  C   CA

                520         *       540         *       560         *       580         *       600         *
AS1 : AGGAAGCCCTGATTGGCCAAGAGGAG-GAAGTGACTGTCACAGATGTTGGACAAAGAGAATCTGAAGATGTGAGTGAAAGGGACTCAGACAAAGAGATGGCT :  609
AS2 : AAGGTGCGCTGTGCGATGATGCTGGCGTCCTCACGATCACAG---TTGGACAAAGAGAATCTGAAGATGTGAGTGAAAGGGACTCAGACAAAGAGATGGCT :  335
      A G  GC CTG   G  A G  G G G   T AC  TCACAG   TTGGACAAAGAGAATCTGAAGATGTGAGTGAAAGGGACTCAGACAAAGAGATGGCT

              620         *       640         *       660         *       680         *       700         *
AS1 : GCTAACTCAGCGGTGGTTCAGGACATCACGAAGGAGGGAAAGGAGGAAATGCCTGAGATGATGGAACCGATCCCTGCCTCGGAAAGCAGCGTGGATGAGCTG :  711
AS2 : GCTAACTCAGCGGTGGTTCAGGACATCACGAAGGAGGGAAAGGAGGAAATGCCTGAGATGATGGAACCGATCCCTGCCTCGGAAAGCAGCGTGGATGAGCTG :  437
      GCTAACTCAGCGGTGGTTCAGGACATCACGAAGGAGGGAAAGGAGGAAATGCCTGAGATGATGGAACCGATCCCTGCCTCGGAAAGCAGCGTGGATGAGCTG

            720         *       740         *       760         *       780         *       800         *
AS1 : ATGCAACCCCCTGAGCCCCAGGCTAATGACGTGGGATTTAAGAAGGTGTTCAAGTTTGTCGGCTTCAAATTCACCGTGAAAAAGGATAAGACCGAGAAGTCT :  813
AS2 : ATGCAACCCCCTGAGCCCCAGGCTAATGACGTGGGATTTAAGAAGGTGTTCAAGTTTGTCGGCTTCAAATTCACCGTGAAAAAGGATAAGACCGAGAAGTCT :  539
      ATGCAACCCCCTGAGCCCCAGGCTAATGACGTGGGATTTAAGAAGGTGTTCAAGTTTGTCGGCTTCAAATTCACCGTGAAAAAGGATAAGACCGAGAAGTCT

          820         *       840         *       860         *       880         *       900         *       9
AS1 : GACAGCGTGCAGCTCCTCACCGTCAAGAAGGATGAAAGTGAAGGGGCAGGCGAGTCCGACGGGGCTGGTGACCACCCGGAGCCCAGCCGGGAGCCGGGGGAT :  915
AS2 : GACAGCGTGCAGCTCCTCACCGTCAAGAAGGATGAAAGTGAAGGGGCAGGCGAGTCCGACGGGGCTGGTGACCACCCGGAGCCCAGCCGGGAGCCGGGGGAT :  641
      GACAGCGTGCAGCTCCTCACCGTCAAGAAGGATGAAAGTGAAGGGGCAGGCGAGTCCGACGGGGCTGGTGACCACCCGGAGCCCAGCCGGGAGCCGGGGGAT

      20         *       940         *       960         *       980         *      1000         *      1020
AS1 : GCGACACCTAAAGACGGTGAACTGAAACAGTCCACCGAGAAACCCGAAGAGGCACCCCGACGTGAGCAGAGCTGCACAGAAATCTCCCTTCAGACCGAGTCT : 1017
AS2 : GCGACACCTAAAGACGGTGAACTGAAACAGTCCACCGAGAAACCCGAAGAGGCACCCCGACGTGAGCAGAGCTGCACAGAAATCTCCCTTCAGACCGAGTCT :  743
      GCGACACCTAAAGACGGTGAACTGAAACAGTCCACCGAGAAACCCGAAGAGGCACCCCGACGTGAGCAGAGCTGCACAGAAATCTCCCTTCAGACCGAGTCT

                *      1040         *      1060         *      1080         *      1100         *      1120
AS1 : GGTCAAGCAGCTGAGGAAGGCAAAGATGAAGGAGAAGAGAAACAAGAGAAAGAACCCGCCAGACCTGCAGACTCCCCGACTAGTCCCGTGGCCAGCGAAACC : 1119
AS2 : GGTCAAGCAGCTGAGGAAGGCAAAGATGAAGGAGAAGAGAAACAAGAGAAAGAACCCGCCAGACCTGCAGACTCCCCGACTAGTCCCGTGGCCAGCGAAACC :  845
      GGTCAAGCAGCTGAGGAAGGCAAAGATGAAGGAGAAGAGAAACAAGAGAAAGAACCCGCCAGACCTGCAGACTCCCCGACTAGTCCCGTGGCCAGCGAAACC

              *      1140         *      1160         *      1180         *      1200         *      1220
AS1 : GCCTCGCCCTTCAGAAAATTCTTCACTCAAGGCTGGGCCGGCTGGCGCAAAAAGACCAGCTTCAGGAAGCCCCGGGAGGAGGAGCTGGAGGCTTCCGAGAAG : 1221
AS2 : GCCTCGCCCTTCAGAAAATTCTTCACTCAAGGCTGGGCCGGCTGGCGCAAAAAGACCAGCTTCAGGAAGCCCCGGGAGGAGGAGCTGGAGGCTTCCGAGAAG :  947
      GCCTCGCCCTTCAGAAAATTCTTCACTCAAGGCTGGGCCGGCTGGCGCAAAAAGACCAGCTTCAGGAAGCCCCGGGAGGAGGAGCTGGAGGCTTCCGAGAAG

            *      1240         *      1260         *      1280         *      1300         *      1320
AS1 : AAAAAGGAACAGGAGCCCGAAAAAGCCGAAGAAAATGAGAAGATGGAAGAGCCCTGCGAGCAGCCACCTGCCCAGGAGGCTCCCGAGAGCACCCAGGATGCC : 1323
AS2 : AAAAAGGAACAGGAGCCCGAAAAAGCCGAAGAAAATGAGAAGATGGAAGAGCCCTGCGAGCAGCCACCTGCCCAGGAGGCTCCCGAGAGCACCCAGGATGCC : 1049
      AAAAAGGAACAGGAGCCCGAAAAAGCCGAAGAAAATGAGAAGATGGAAGAGCCCTGCGAGCAGCCACCTGCCCAGGAGGCTCCCGAGAGCACCCAGGATGCC

          *      1340         *      1360         *      1380         *      1400         *      1420
AS1 : AGGCTCTCGGCCGAGTATGAAAAAGTGGAGCTGCCCCCTGAAGACCAAGTGCAGGCACCTCCTGAAGAGAAACCAGCCCCGTTAGCAACCGAAGTGTTTGAT : 1425
AS2 : AGGCTCTCGGCCGAGTATGAAAAAGTGGAGCTGCCCCCTGAAGACCAAGTGCAGGCACCTCCTGAAGAGAAACCAGCCCCGTTAGCAACCGAAGTGTTTGAT : 1151
      AGGCTCTCGGCCGAGTATGAAAAAGTGGAGCTGCCCCCTGAAGACCAAGTGCAGGCACCTCCTGAAGAGAAACCAGCCCCGTTAGCAACCGAAGTGTTTGAT

        *      1440         *      1460         *      1480         *      1500         *      1520         *
AS1 : GAAAAGGTCGAGATTGTCGCCGAAGTCCACGTTAGCACTGTAGAGAAGGACCAGGAAGAGCCGAAAACCGAAGCGGAAGAAACAGTGGAGCTCTCGCTGCCC : 1527
AS2 : GAAAAGGTCGAGATTGTCGCCGAAGTCCACGTTAGCACTGTAGAGAAGGACCAGGAAGAGCCGAAAACCGAAGCGGAAGAAACAGTGGAGCTCTCGCTGCCC : 1253
      GAAAAGGTCGAGATTGTCGCCGAAGTCCACGTTAGCACTGTAGAGAAGGACCAGGAAGAGCCGAAAACCGAAGCGGAAGAAACAGTGGAGCTCTCGCTGCCC

             1540         *      1560         *      1580         *      1600         *      1620         *
AS1 : GAAAAGTCGACCGAGACGAGCGCCGACCTTCCGGAAGCCGAGTCAGCCGAGATGCTGAAGACAGAGGAAGATGCGGGCGCCCTGAGCGGGGACCACTCCCAG : 1629
AS2 : GAAAAGTCGACCGAGACGAGCGCCGACCTTCCGGAAGCCGAGTCAGCCGAGATGCTGAAGACAGAGGAAGATGCGGGCGCCCTGAGCGGGGACCACTCCCAG : 1355
      GAAAAGTCGACCGAGACGAGCGCCGACCTTCCGGAAGCCGAGTCAGCCGAGATGCTGAAGACAGAGGAAGATGCGGGCGCCCTGAGCGGGGACCACTCCCAG

           1640         *      1660         *      1680         *      1700         *      1720         *
AS1 : GCAACCGAGCTCAGCGGGGAGGCAAAATCGCCGTCCACGCCCCCCGAGGGCACCGTGAGGGAGGCCGAAATGCTGTCCTCGCAGGAGAGAATTAAGGTGCAG : 1731
AS2 : GCAACCGAGCTCAGCGGGGAGGCAAAATCGCCGTCCACGCCCCCCGAGGGCACCGTGAGGGAGGCCGAAATGCTGTCCTCGCAGGAGAGAATTAAGGTGCAG : 1457
      GCAACCGAGCTCAGCGGGGAGGCAAAATCGCCGTCCACGCCCCCCGAGGGCACCGTGAGGGAGGCCGAAATGCTGTCCTCGCAGGAGAGAATTAAGGTGCAG

         1740         *      1760         *      1780         *      1800         *      1820         *
AS1 : GGAAGCCCTTTGAAGAAACTGTTCACGAGCACCGGCTTGAAAAAGCTCTCTGGAAAGAAACAGAAAGGGAAACGAGGCGGCGACGAGGAGTCCGGGGAGCAG : 1833
AS2 : GGAAGCCCTTTGAAGAAACTGTTCACGAGCACCGGCTTGAAAAAGCTCTCTGGAAAGAAACAGAAAGGGAAACGAGGCGGCGACGAGGAGTCCGGGGAGCAG : 1559
      GGAAGCCCTTTGAAGAAACTGTTCACGAGCACCGGCTTGAAAAAGCTCTCTGGAAAGAAACAGAAAGGGAAACGAGGCGGCGACGAGGAGTCCGGGGAGCAG

      1840         *      1860         *      1880         *      1900         *      1920         *       19
AS1 : CCCCCAGCCTCCGTGGATTCTCTCGACAGTCCAGACGAACCGAAGGGCGGCGAGAGCTCAGCCTCGTCTCCAGAAGAGCCCGAGGAGATCACCTGTCTGGAG : 1935
AS2 : CCCCCAGCCTCCGTGGATTCTCTCGACAGTCCAGACGAACCGAAGGGCGGCGAGAGCTCAGCCTCGTCTCCAGAAGAGCCCGAGGAGATCACCTGTCTGGAG : 1661
      CCCCCAGCCTCCGTGGATTCTCTCGACAGTCCAGACGAACCGAAGGGCGGCGAGAGCTCAGCCTCGTCTCCAGAAGAGCCCGAGGAGATCACCTGTCTGGAG

      40         *      1960         *      1980         *      2000         *      2020         *      2040
AS1 : AAAGAGGTGGCCGAGGCCCCGGGCCAGGAGGGGGAGGTGGAAGAAGGGACCACTTCCGACGGGGAGAAGAAGAGGGAAGGTGGTGTCACCCCCTGGGCGTCT : 2037
AS2 : AAAGAGGTGGCCGAGGCCCCGGGCCAGGAGGGGGAGGTGGAAGAAGGGACCACTTCCGACGGGGAGAAGAAGAGGGAAGGTGGTGTCACCCCCTGGGCGTCT : 1763
      AAAGAGGTGGCCGAGGCCCCGGGCCAGGAGGGGGAGGTGGAAGAAGGGACCACTTCCGACGGGGAGAAGAAGAGGGAAGGTGGTGTCACCCCCTGGGCGTCT

                *      2060         *      2080         *      2100         *      2120         *      2140
AS1 : TTCAAAAAGATGGTGACGCCCAAGAAACGCGTGCGAAGGCTCTCTGAAAGCGATAAGGAGGACGAGGCAGACAAAGTCAAAAGCGCCACCCTGTCCTCCACG : 2139
AS2 : TTCAAAAAGATGGTGACGCCCAAGAAACGCGTGCGAAGGCTCTCTGAAAGCGATAAGGAGGACGAGGCAGACAAAGTCAAAAGCGCCACCCTGTCCTCCACG : 1865
      TTCAAAAAGATGGTGACGCCCAAGAAACGCGTGCGAAGGCTCTCTGAAAGCGATAAGGAGGACGAGGCAGACAAAGTCAAAAGCGCCACCCTGTCCTCCACG
```

```
             *      2160         *      2180         *      2200         *      2220         *      2240
AS1 : GAGAGCGCGGCCTCTGAAGTGCAGGAAGAGGCCAAAGGGAACGGAGAGGAGCCTAAGCCCGAGGAGCCCAAACGCAAGGTCGACACCTCCGTGTCCTGGGAA : 2241
AS2 : GAGAGCGCGGCCTCTGAAGTGCAGGAAGAGGCCAAAGGGAACGGAGAGGAGCCTAAGCCCGAGGAGCCCAAACGCAAGGTCGACACCTCCGTGTCCTGGGAA : 1967
      GAGAGCGCGGCCTCTGAAGTGCAGGAAGAGGCCAAAGGGAACGGAGAGGAGCCTAAGCCCGAGGAGCCCAAACGCAAGGTCGACACCTCCGTGTCCTGGGAA

           *      2260         *      2280         *      2300         *      2320         *      2340
AS1 : GCCCTGATTTGCGTGGGGTCATCCAAGAAGAGAGCCAGAAAAGCATCCTCCTCCGATGATGAAGCAGGGCCGAAACCTCTCGGAGGGGACAGCCAGAGAGCA : 2343
AS2 : GCCCTGATTTGCGTGGGGTCATCCAAGAAGAGAGCCAGAAAAGCATCCTCCTCCGATGATGAAGCAGGGCCGAAACCTCTCGGAGGGGACAGCCAGAGAGCA : 2069
      GCCCTGATTTGCGTGGGGTCATCCAAGAAGAGAGCCAGAAAAGCATCCTCCTCCGATGATGAAGCAGGGCCGAAACCTCTCGGAGGGGACAGCCAGAGAGCA

         *      2360         *      2380         *      2400         *      2420         *      2440
AS1 : GAGGAAGCTGGGAAGGACAAGGAGCCGGGCCCGGAGGCGGCCGCTGCCGCTTCCCAGGACCACGAGCAACCGCCGGGAAGCTCCTCACCCGAGCCGGCCGGC : 2445
AS2 : GAGGAAGCTGGGAAGGACAAGGAGCCGGGCCCGGAGGCGGCCGCTGCCGCTTCCCAGGACCACGAGCAACCGCCGGGAAGCTCCTCACCCGAGCCGGCCGGC : 2171
      GAGGAAGCTGGGAAGGACAAGGAGCCGGGCCCGGAGGCGGCCGCTGCCGCTTCCCAGGACCACGAGCAACCGCCGGGAAGCTCCTCACCCGAGCCGGCCGGC

       *      2460         *      2480         *      2500         *      2520         *      2540         *
AS1 : AGCCCATCCGAAGGGGAGGGCGTCTCCACCTGGGAGACCTTTAAAAGACTGGTCACCTCGAGAAAAAAATCGAAGTCAAAACTGGAAGAGAGAAGTGAAGAC : 2547
AS2 : AGCCCATCCGAAGGGGAGGGCGTCTCCACCTGGGAGACCTTTAAAAGACTGGTCACCTCGAGAAAAAAATCGAAGTCAAAACTGGAAGAGAGAAGTGAAGAC : 2273
      AGCCCATCCGAAGGGGAGGGCGTCTCCACCTGGGAGACCTTTAAAAGACTGGTCACCTCGAGAAAAAAATCGAAGTCAAAACTGGAAGAGAGAAGTGAAGAC

            2560         *      2580         *      2600         *      2620         *      2640         *
AS1 : TCTGTAGCTGGGTCTGGCGCAGAACATGCAGCCTCAGAGGCTGAGCCTGGGAAAGAAGAGTCCTGGGTTTCCATCCGGAAGTTTATTCCCGGGCGAAGGAAG : 2649
AS2 : TCTGTAGCTGGGTCTGGCGCAGAACATGCAGCCTCAGAGGCTGAGCCTGGGAAAGAAGAGTCCTGGGTTTCCATCCGGAAGTTTATTCCCGGGCGAAGGAAG : 2375
      TCTGTAGCTGGGTCTGGCGCAGAACATGCAGCCTCAGAGGCTGAGCCTGGGAAAGAAGAGTCCTGGGTTTCCATCCGGAAGTTTATTCCCGGGCGAAGGAAG

          2660         *      2680         *      2700         *      2720         *      2740         *
AS1 : AAAAGGCCAGATGGGAAGCAGGAGCCAGCCGCCGTCGAAGAGGCGGGGCCAGCGGAGGCCAACGAGGACGATGCGGACATCCCTGCCGTGGTCCCTCTGTCT : 2751
AS2 : AAAAGGCCAGATGGGAAGCAGGAGCCAGCCGCCGTCGAAGAGGCGGGGCCAGCGGAGGCCAACGAGGACGATGCGGACATCCCTGCCGTGGTCCCTCTGTCT : 2477
      AAAAGGCCAGATGGGAAGCAGGAGCCAGCCGCCGTCGAAGAGGCGGGGCCAGCGGAGGCCAACGAGGACGATGCGGACATCCCTGCCGTGGTCCCTCTGTCT

        2760         *      2780         *      2800         *      2820         *      2840         *
AS1 : GAATACGACGCGGTGGAAAGAGAGAAAACCGAAGCCCAGCAAGCCCCCAAGAGCCAGGAGGGGCCCGAGCAGAAGCAGGTGGATGTCGACGTGTCAGAGGAG : 2853
AS2 : GAATACGACGCGGTGGAAAGAGAGAAAACCGAAGCCCAGCAAGCCCCCAAGAGCCAGGAGGGGCCCGAGCAGAAGCAGGTGGATGTCGACGTGTCAGAGGAG : 2579
      GAATACGACGCGGTGGAAAGAGAGAAAACCGAAGCCCAGCAAGCCCCCAAGAGCCAGGAGGGGCCCGAGCAGAAGCAGGTGGATGTCGACGTGTCAGAGGAG

      2860         *      2880         *      2900         *      2920         *      2940         *      29
AS1 : CTCAGTAAGAGCCTGGTTCACACCGTGTCGGTGGCTGTCGTGGACGGGACAAGGGCCATTACCAACATCGAAGAAAGGGCACCCTCCTGGATCTCAGCTTCG : 2955
AS2 : CTCAGTAAGAGCCTGGTTCACACCGTGTCGGTGGCTGTCGTGGACGGGACAAGGGCCATTACCAACATCGAAGAAAGGGCACCCTCCTGGATCTCAGCTTCG : 2681
      CTCAGTAAGAGCCTGGTTCACACCGTGTCGGTGGCTGTCGTGGACGGGACAAGGGCCATTACCAACATCGAAGAAAGGGCACCCTCCTGGATCTCAGCTTCG

      60         *      2980         *      3000         *      3020         *      3040         *      3060
AS1 : GTGACAGAACCACTGGAACAAGCCGAAGGCGAAGCCACACCACCAAGCGAGGAGGTGCTTGAAAGAGAAGTCGTCGTCGCAGAGGAAACCCCCATCGTTTCC : 3057
AS2 : GTGACAGAACCACTGGAACAAGCCGAAGGCGAAGCCACACCACCAAGCGAGGAGGTGCTTGAAAGAGAAGTCGTCGTCGCAGAGGAAACCCCCATCGTTTCC : 2783
      GTGACAGAACCACTGGAACAAGCCGAAGGCGAAGCCACACCACCAAGCGAGGAGGTGCTTGAAAGAGAAGTCGTCGTCGCAGAGGAAACCCCCATCGTTTCC

               *      3080         *      3100         *      3120         *      3140         *      3160
AS1 : AAAGCACTGCCAGAGGTCCGGGAGGCCAGTGATGACACGATGGCCAGCGAGGTGGAATTAACCTCGGAAGCTGTGACAGCCGCCGAAACCACAGAGGCCTCT : 3159
AS2 : AAAGCACTGCCAGAGGTCCGGGAGGCCAGTGATGACACGATGGCCAGCGAGGTGGAATTAACCTCGGAAGCTGTGACAGCCGCCGAAACCACAGAGGCCTCT : 2885
      AAAGCACTGCCAGAGGTCCGGGAGGCCAGTGATGACACGATGGCCAGCGAGGTGGAATTAACCTCGGAAGCTGTGACAGCCGCCGAAACCACAGAGGCCTCT

             *      3180         *      3200         *      3220         *      3240         *      3260
AS1 : GGTGCGGAAGAAACCGCCGACATGGTTTCCGCTGTCTCTCAGTTAACCGACTCTCCAGACACCACCGAGGAAGCGACACCAGTGCAAGAGGTGGAGGGCGGC : 3261
AS2 : GGTGCGGAAGAAACCGCCGACATGGTTTCCGCTGTCTCTCAGTTAACCGACTCTCCAGACACCACCGAGGAAGCGACACCAGTGCAAGAGGTGGAGGGCGGC : 2987
      GGTGCGGAAGAAACCGCCGACATGGTTTCCGCTGTCTCTCAGTTAACCGACTCTCCAGACACCACCGAGGAAGCGACACCAGTGCAAGAGGTGGAGGGCGGC

           *      3280         *      3300         *      3320         *      3340         *      3360
AS1 : GTGCCTGACGCAGAGGACCAGGCGAGAAGGACCCAAGAGGTGCTGCAGGCCGTTGCAGAAAAAGTTAGAGAAGAGTCACAGCTGCCAGATGACGCCATCCAG : 3363
AS2 : GTGCCTGACGCAGAGGACCAGGCGAGAAGGACCCAAGAGGTGCTGCAGGCCGTTGCAGAAAAAGTTAGAGAAGAGTCACAGCTGCCAGATGACGCCATCCAG : 3089
      GTGCCTGACGCAGAGGACCAGGCGAGAAGGACCCAAGAGGTGCTGCAGGCCGTTGCAGAAAAAGTTAGAGAAGAGTCACAGCTGCCAGATGACGCCATCCAG

         *      3380         *      3400         *      3420         *      3440         *      3460
AS1 : AAAGGACAAGCAAAAATACTGGAGAAAGTGGAAGAGGCTGAGGAGGGTTCTCAGGCCCTAGATCTGAAGGAAATGATGGATGTAGCATCCAACGTGCTTGTC : 3465
AS2 : AAAGGACAAGCAAAAATACTGGAGAAAGTGGAAGAGGCTGAGGAGGGTTCTCAGGCCCTAGATCTGAAGGAAATGATGGATGTAGCATCCAACGTGCTTGTC : 3191
      AAAGGACAAGCAAAAATACTGGAGAAAGTGGAAGAGGCTGAGGAGGGTTCTCAGGCCCTAGATCTGAAGGAAATGATGGATGTAGCATCCAACGTGCTTGTC

       *      3480         *      3500         *      3520         *      3540         *      3560         *
AS1 : CAAGGTACTGAAACTGAGACTTTGACACAGGAGAAGGTGGTTGGAGAGGCCACCGTGGAAAGCTTGGGAGAAGTTCCTCCAGGCACAGACAGTGCAGAGGCC : 3567
AS2 : CAAGGTACTGAAACTGAGACTTTGACACAGGAGAAGGTGGTTGGAGAGGCCACCGTGGAAAGCTTGGGAGAAGTTCCTCCAGGCACAGACAGTGCAGAGGCC : 3293
      CAAGGTACTGAAACTGAGACTTTGACACAGGAGAAGGTGGTTGGAGAGGCCACCGTGGAAAGCTTGGGAGAAGTTCCTCCAGGCACAGACAGTGCAGAGGCC

            3580         *      3600         *      3620         *      3640         *      3660         *
AS1 : GGGGAGCTGGCGAGCACTTGTCTGGCTGAAACCGGGACTGGGGTAGAAGCAGAGACTGCCCCAGAGCAGGCTGTTGCTCCTGACTCAGCTGAAACCCTCACG : 3669
AS2 : GGGGAGCTGGCGAGCACTTGTCTGGCTGAAACCGGGACTGGGGTAGAAGCAGAGACTGCCCCAGAGCAGGCTGTTGCTCCTGACTCAGCTGAAACCCTCACG : 3395
      GGGGAGCTGGCGAGCACTTGTCTGGCTGAAACCGGGACTGGGGTAGAAGCAGAGACTGCCCCAGAGCAGGCTGTTGCTCCTGACTCAGCTGAAACCCTCACG

          3680         *      3700         *      3720         *      3740         *      3760         *
AS1 : GACAGTGAGATCAATGGAAGCACCCCAGTAGCAGATCTTGATGCTTCAAATCTAAGCCAGCCAGACAAGATCATGGATGTCCGTGAAGATCATGAGGTTCCA : 3771
AS2 : GACAGTGAGATCAATGGAAGCACCCCAGTAGCAGATCTTGATGCTTCAAATCTAAGCCAGCCAGACAAGATCATGGATGTCCGTGAAGATCATGAGGTTCCA : 3497
      GACAGTGAGATCAATGGAAGCACCCCAGTAGCAGATCTTGATGCTTCAAATCTAAGCCAGCCAGACAAGATCATGGATGTCCGTGAAGATCATGAGGTTCCA

        3780         *      3800         *      3820         *      3840         *      3860         *
AS1 : GCTAGCACTCAGTCCCAGGTCCCCGAAGGCGAGGTGCCTCCTGCACTGAAAGAGCTGCCTGCAGCATCTTCTGATTTTCAGTCGCAGGAGGAAAGGTCTTCA : 3873
AS2 : GCTAGCACTCAGTCCCAGGTCCCCGAAGGCGAGGTGCCTCCTGCACTGAAAGAGCTGCCTGCAGCATCTTCTGATTTTCAGTCGCAGGAGGAAAGGTCTTCA : 3599
      GCTAGCACTCAGTCCCAGGTCCCCGAAGGCGAGGTGCCTCCTGCACTGAAAGAGCTGCCTGCAGCATCTTCTGATTTTCAGTCGCAGGAGGAAAGGTCTTCA

      3880         *      3900         *      3920         *      3940         *      3960         *      39
AS1 : AAGATGACAGAGGGTCTAGAACACACGGATGAAGAGGAGGGAACAGTGGAGACTGTAGCCATCCTTTCAAAGACTGAGGTCATTCAAGAGACTGGCCAGTGC : 3975
AS2 : AAGATGACAGAGGGTCTAGAACACACGGATGAAGAGGAGGGAACAGTGGAGACTGTAGCCATCCTTTCAAAGACTGAGGTCATTCAAGAGACTGGCCAGTGC : 3701
      AAGATGACAGAGGGTCTAGAACACACGGATGAAGAGGAGGGAACAGTGGAGACTGTAGCCATCCTTTCAAAGACTGAGGTCATTCAAGAGACTGGCCAGTGC

      80         *      4000         *      4020         *      4040         *      4060         *      4080
AS1 : TCTGACGAGGAAGCCAAAGAAGAACCATCCATGGAAGGACTTGCCGTGTCTGCCGACACAGAAATAACTGAGAAAAAGATAACTGAAGTTGTCCTTGAGGAG : 4077
AS2 : TCTGACGAGGAAGCCAAAGAAGAACCATCCATGGAAGGACTTGCCGTGTCTGCCGACACAGAAATAACTGAGAAAAAGATAACTGAAGTTGTCCTTGAGGAG : 3803
      TCTGACGAGGAAGCCAAAGAAGAACCATCCATGGAAGGACTTGCCGTGTCTGCCGACACAGAAATAACTGAGAAAAAGATAACTGAAGTTGTCCTTGAGGAG

               *      4100         *      4120         *      4140         *      4160         *      4180
AS1 : GACGTTACTAAGAAAGTTGAATTTCAAGAGAATGAAAATATAGAACTCCAGAGTCCTGCTAAGTTTCTTCCAACCCCAGAAGAGAGAGAGTTGGTAGTTGAA : 4179
AS2 : GACGTTACTAAGAAAGTTGAATTTCAAGAGAATGAAAATATAGAACTCCAGAGTCCTGCTAAGTTTCTTCCAACCCCAGAAGAGAGAGAGTTGGTAGTTGAA : 3905
      GACGTTACTAAGAAAGTTGAATTTCAAGAGAATGAAAATATAGAACTCCAGAGTCCTGCTAAGTTTCTTCCAACCCCAGAAGAGAGAGAGTTGGTAGTTGAA

             *      4200         *      4220         *      4240         *      4260         *      4280
AS1 : GGGAAAAGGGAGACTGTGGAAGTGGAGGCAACTGAAGGGAATGAAGAGAAACTTGAGCACGAACCAGCTGTGGCCGTATGTGAAGAGCTCAGTAAGCAACTG : 4281
AS2 : GGGAAAAGGGAGACTGTGGAAGTGGAGGCAACTGAAGGGAATGAAGAGAAACTTGAGCACGAACCAGCTGTGGCCGTATGTGAAGAGCTCAGTAAGCAACTG : 4007
      GGGAAAAGGGAGACTGTGGAAGTGGAGGCAACTGAAGGGAATGAAGAGAAACTTGAGCACGAACCAGCTGTGGCCGTATGTGAAGAGCTCAGTAAGCAACTG
```

```
             *       4300         *       4320         *       4340         *       4360         *       4380
AS1 : GTTCAGACAGTGAACGTCACCATCATCGACGGGGAAAAGGAAGTCGTCAGTTTTGAAGGAAGTTCTCCCCTGCTGGCTTCCGAGGAAGAGGCATGCACGGGA : 4383
AS2 : GTTCAGACAGTGAACGTCACCATCATCGACGGGGAAAAGGAAGTCGTCAGTTTTGAAGGAAGTTCTCCCCTGCTGGCTTCCGAGGAAGAGGCATGCACGGGA : 4109
      GTTCAGACAGTGAACGTCACCATCATCGACGGGGAAAAGGAAGTCGTCAGTTTTGAAGGAAGTTCTCCCCTGCTGGCTTCCGAGGAAGAGGCATGCACGGGA

           *       4400         *       4420         *       4440         *       4460         *       4480
AS1 : ATCTCAGTTCAAAGCTCTGAGGCAGCATTAACTCTGATAGCTGCAGCCGTGGAGGAGAAGGTCTTAGGAGAAGCTATCAAGATTGTAGAAACAACCGAGGCT : 4485
AS2 : ATCTCAGTTCAAAGCTCTGAGGCAGCATTAACTCTGATAGCTGCAGCCGTGGAGGAGAAGGTCTTAGGAGAAGCTATCAAGATTGTAGAAACAACCGAGGCT : 4211
      ATCTCAGTTCAAAGCTCTGAGGCAGCATTAACTCTGATAGCTGCAGCCGTGGAGGAGAAGGTCTTAGGAGAAGCTATCAAGATTGTAGAAACAACCGAGGCT

         *       4500         *       4520         *       4540         *       4560         *       4580         *
AS1 : TTGAAATCTGCAGAAGCTCATCTAGTCCTGGAAGAAGAGTCCTCAGAAAAAGATGAAGAGTTTCCTGCCCAGCCTGGGGAAGAAGATGTGGCATCCACAGGG : 4587
AS2 : TTGAAATCTGCAGAAGCTCATCTAGTCCTGGAAGAAGAGTCCTCAGAAAAAGATGAAGAGTTTCCTGCCCAGCCTGGGGAAGAAGATGTGGCATCCACAGGG : 4313
      TTGAAATCTGCAGAAGCTCATCTAGTCCTGGAAGAAGAGTCCTCAGAAAAAGATGAAGAGTTTCCTGCCCAGCCTGGGGAAGAAGATGTGGCATCCACAGGG

               4600         *       4620         *       4640         *       4660         *       4680         *
AS1 : ACTGAGTCTCAAGCAGAATCAATACTGACGATAGTATCTGTTACGCCTGGAAAAGGCATCAGCGCTGACCTGGAAGGAGACGAAACCGTGTCACAGAAATGG : 4689
AS2 : ACTGAGTCTCAAGCAGAATCAATACTGACGATAGTATCTGTTACGCCTGGAAAAGGCATCAGCGCTGACCTGGAAGGAGACGAAACCGTGTCACAGAAATGG : 4415
      ACTGAGTCTCAAGCAGAATCAATACTGACGATAGTATCTGTTACGCCTGGAAAAGGCATCAGCGCTGACCTGGAAGGAGACGAAACCGTGTCACAGAAATGG

             4700         *       4720         *       4740         *       4760         *       4780         *
AS1 : GAGTTGGGTGGAGACGGCAAGCAGGCTGGTGGTCAGGAAGGCAGAGAGAGCAACGCAGGAGAGGAAGACGGCAAGGCGGAAAGTGAGATTTTGAAGCTTGAG : 4791
AS2 : GAGTTGGGTGGAGACGGCAAGCAGGCTGGTGGTCAGGAAGGCAGAGAGAGCAACGCAGGAGAGGAAGACGGCAAGGCGGAAAGTGAGATTTTGAAGCTTGAG : 4517
      GAGTTGGGTGGAGACGGCAAGCAGGCTGGTGGTCAGGAAGGCAGAGAGAGCAACGCAGGAGAGGAAGACGGCAAGGCGGAAAGTGAGATTTTGAAGCTTGAG

         4800         *       4820         *       4840         *       4860         *       4880         *
AS1 : AAGGAGAGCTGCAGACTTGTACAGAGCGTGATTCAGACAGCCGTTGACCGTTTGGGGAGTACAGAAGAAACAGCCACCGATGTCCAGACTCAGGCTCAACTG : 4893
AS2 : AAGGAGAGCTGCAGACTTGTACAGAGCGTGATTCAGACAGCCGTTGACCGTTTGGGGAGTACAGAAGAAACAGCCACCGATGTCCAGACTCAGGCTCAACTG : 4619
      AAGGAGAGCTGCAGACTTGTACAGAGCGTGATTCAGACAGCCGTTGACCGTTTGGGGAGTACAGAAGAAACAGCCACCGATGTCCAGACTCAGGCTCAACTG

      4900         *       4920         *       4940         *       4960         *       4980         *       50
AS1 : GCAGAAGTCGACAGCCAGGAAGCTGGGCAGAAAACGGAGAAAGAAGAAAGTGAACCTCAGGCCTGCCCGGTGGATGAAACACAAGCCGTAGAAGCCAAAGAC : 4995
AS2 : GCAGAAGTCGACAGCCAGGAAGCTGGGCAGAAAACGGAGAAAGAAGAAAGTGAACCTCAGGCCTGCCCGGTGGATGAAACACAAGCCGTAGAAGCCAAAGAC : 4721
      GCAGAAGTCGACAGCCAGGAAGCTGGGCAGAAAACGGAGAAAGAAGAAAGTGAACCTCAGGCCTGCCCGGTGGATGAAACACAAGCCGTAGAAGCCAAAGAC

      00         *       5020         *       5040         *       5060         *       5080         *       5100
AS1 : GAGTCCCCACTAAGCACCGGGGAACACGCGCATCTCAGCGTTTGCAAAGATGCAATTGAAGCTTCAGAGAAGGTGGCGGCCACTAGCATAGAAGGTTCCAGG : 5097
AS2 : GAGTCCCCACTAAGCACCGGGGAACACGCGCATCTCAGCGTTTGCAAAGATGCAATTGAAGCTTCAGAGAAGGTGGCGGCCACTAGCATAGAAGGTTCCAGG : 4823
      GAGTCCCCACTAAGCACCGGGGAACACGCGCATCTCAGCGTTTGCAAAGATGCAATTGAAGCTTCAGAGAAGGTGGCGGCCACTAGCATAGAAGGTTCCAGG

               *       5120         *       5140         *       5160         *       5180         *       5200
AS1 : GTAGAAGACCAGTGGCTTGAAGAGGTAGCTCTCCCATCCCAGAAAAAGAGAGAAATCCCTGAAACAGAGTCTGTGCTGCAAGATGACGGCAGTGCCGGGTTT : 5199
AS2 : GTAGAAGACCAGTGGCTTGAAGAGGTAGCTCTCCCATCCCAGAAAAAGAGAGAAATCCCTGAAACAGAGTCTGTGCTGCAAGATGACGGCAGTGCCGGGTTT : 4925
      GTAGAAGACCAGTGGCTTGAAGAGGTAGCTCTCCCATCCCAGAAAAAGAGAGAAATCCCTGAAACAGAGTCTGTGCTGCAAGATGACGGCAGTGCCGGGTTT

             *       5220         *       5240         *       5260         *       5280         *       5300
AS1 : GGAGAAAGAAAGAAGTCACCATTTGAATCCCGAGAAGATGAAAAAGGTGATGCTGCCGGTGACCCTGGAAACCAAACCTCAACCCCAGAGGATGCTGAGGCC : 5301
AS2 : GGAGAAAGAAAGAAGTCACCATTTGAATCCCGAGAAGATGAAAAAGGTGATGCTGCCGGTGACCCTGGAAACCAAACCTCAACCCCAGAGGATGCTGAGGCC : 5027
      GGAGAAAGAAAGAAGTCACCATTTGAATCCCGAGAAGATGAAAAAGGTGATGCTGCCGGTGACCCTGGAAACCAAACCTCAACCCCAGAGGATGCTGAGGCC

             *       5320         *       5340         *       5360         *       5380         *       5400
AS1 : TCGGGAGGCTCGACCAAAGAGTCCCTAGATACAAATGGACCCAAACTAAAAGAGAAGGGAGACAGCCAGGAGGAAAAAGTGCAGAGCGAGTCAGAAAAAGAG : 5403
AS2 : TCGGGAGGCTCGACCAAAGAGTCCCTAGATACAAATGGACCCAAACTAAAAGAGAAGGGAGACAGCCAGGAGGAAAAAGTGCAGAGCGAGTCAGAAAAAGAG : 5129
      TCGGGAGGCTCGACCAAAGAGTCCCTAGATACAAATGGACCCAAACTAAAAGAGAAGGGAGACAGCCAGGAGGAAAAAGTGCAGAGCGAGTCAGAAAAAGAG

           *       5420         *       5440         *       5460         *       5480         *       5500
AS1 : ATGAAAATACAAACACAGGAGGAGACACAGAACCAAGAGAGAGATCTGGCGAAACCTGAGCCCACGGAATCCTAAAACATCACTTACACTTGTGTACTTGCA : 5505
AS2 : ATGAAAATACAAACACAGGAGGAGACACAGAACCAAGAGAGAGATCTGGCGAAACCTGAGCCCACGGAATCCTAAAACATCACTTACACTTGTGTACTTGCA : 5231
      ATGAAAATACAAACACAGGAGGAGACACAGAACCAAGAGAGAGATCTGGCGAAACCTGAGCCCACGGAATCCTAAAACATCACTTACACTTGTGTACTTGCA

         *       5520         *       5540         *       5560         *       5580         *       5600         *
AS1 : AGACCAGAATGTGAAGACAAGTAGGGGAAGAGATAGAATGCTGCTGACGAGACCTTTGGAAACGGAAGAACAAACATGTCAACTCCTCATTTCCCGAGAACC : 5607
AS2 : AGACCAGAATGTGAAGACAAGTAGGGGAAGAGATAGAATGCTGCTGACGAGACCTTTGGAAACGGAAGAACAAACATGTCAACTCCTCATTTCCCGAGAACC : 5333
      AGACCAGAATGTGAAGACAAGTAGGGGAAGAGATAGAATGCTGCTGACGAGACCTTTGGAAACGGAAGAACAAACATGTCAACTCCTCATTTCCCGAGAACC

               5620         *       5640         *       5660         *       5680         *       5700         *
AS1 : CCTGACAATCCTGAGGCTTCATCGGGAGCTATAGCCAGATAACACTTCCTCTTGTCAAGACCACACTGACATTTTCCCTCAGTACCATATAATGTTTCTGAT : 5709
AS2 : CCTGACAATCCTGAGGCTTCATCGGGAGCTATAGCCAGATAACACTTCCTCTTGTCAAGACCACACTGACATTTTCCCTCAGTACCATATAATGTTTCTGAT : 5435
      CCTGACAATCCTGAGGCTTCATCGGGAGCTATAGCCAGATAACACTTCCTCTTGTCAAGACCACACTGACATTTTCCCTCAGTACCATATAATGTTTCTGAT

             5720         *       5740         *       5760         *       5780         *       5800         *
AS1 : TCAAGGTCCTCAGTTCTTAGCCTGGGAACTGGAGTTGGCAATTCTTGGTTCTGCTTCTCAAACTGGAGTATCATTCTTTATGTATTTATATGTATGATTCAA : 5811
AS2 : TCAAGGTCCTCAGTTCTTAGCCTGGGAACTGGAGTTGGCAATTCTTGGTTCTGCTTCTCAAACTGGAGTATCATTCTTTATGTATTTATATGTATGATTCAA : 5537
      TCAAGGTCCTCAGTTCTTAGCCTGGGAACTGGAGTTGGCAATTCTTGGTTCTGCTTCTCAAACTGGAGTATCATTCTTTATGTATTTATATGTATGATTCAA

        5820         *       5840         *       5860         *       5880         *       5900         *
AS1 : GTAACCCTCCTTCTGTATCTATTGTATAGTTTTTTTTTTTCTTAACATCTAAGGAAATGTACAGCGTAGTGCAATTCCTTTTGTATCCCTTATTATATGACG : 5913
AS2 : GTAACCCTCCTTCTGTATCTATTGTATAGTTTTTTTTTTTCTTAACATCTAAGGAAATGTACAGCGTAGTGCAATTCCTTTTGTATCCCTTATTATATGACG : 5639
      GTAACCCTCCTTCTGTATCTATTGTATAGTTTTTTTTTTTCTTAACATCTAAGGAAATGTACAGCGTAGTGCAATTCCTTTTGTATCCCTTATTATATGACG

      5920         *       5940         *       5960         *       5980         *       6000         *       60
AS1 : GGGCATGCGGCCACGGTGAAGTCTCGGGAAGCTTTCTGCTGCCTCAGTTACAGCCTTTGAAAACAGCTCTCTAGAAATAACATTCCTGATCAGAAGCTCCAC : 6015
AS2 : GGGCATGCGGCCACGGTGAAGTCTCGGGAAGCTTTCTGCTGCCTCAGTTACAGCCTTTGAAAACAGCTCTCTAGAAATAACATTCCTGATCAGAAGCTCCAC : 5741
      GGGCATGCGGCCACGGTGAAGTCTCGGGAAGCTTTCTGCTGCCTCAGTTACAGCCTTTGAAAACAGCTCTCTAGAAATAACATTCCTGATCAGAAGCTCCAC

      20         *       6040         *       6060         *       6080         *       6100         *       6120
AS1 : CTCTTTTAAAATTCACTAAGGATTAGGTTCCATGTTCAGTATCACTCTGAAATAGGTCACTTTCCTATTATGCATAGTATGCTAAAAATAAAAAGGTGGTTT : 6117
AS2 : CTCTTTTAAAATTCACTAAGGATTAGGTTCCATGTTCAGTATCACTCTGAAATAGGTCACTTTCCTATTATGCATAGTATGCTAAAAATAAAAAGGTGGTTT : 5843
      CTCTTTTAAAATTCACTAAGGATTAGGTTCCATGTTCAGTATCACTCTGAAATAGGTCACTTTCCTATTATGCATAGTATGCTAAAAATAAAAAGGTGGTTT

               *       6140         *       6160         *       6180         *       6200         *       6220
AS1 : TTTTTTTGTGGGGGGGTTTGGGGGAGACATACAGAATGTCCCATTGTTACTGGGAAATTTTTCTTCCTAGCCCCTGGGTGGAGGTTGGAAGGAAGTTTTTT : 6219
AS2 : TTTTTTTGTGGGGGGGTTTGGGGGAGACATACAGAATGTCCCATTGTTACTGGGAAATTTTTCTTCCTAGCCCCTGGGTGGAGGTTGGAAGGAAGTTTTTT : 5945
      TTTTTTTGTGGGGGGGTTTGGGGGAGACATACAGAATGTCCCATTGTTACTGGGAAATTTTTCTTCCTAGCCCCTGGGTGGAGGTTGGAAGGAAGTTTTTT

             *       6240         *       6260         *       6280         *       6300         *       6320
AS1 : TCAGGAGCAGATTAAGTTGGAGTCTTTTCTCCTAATTGTTATGGTTGTTTGGACGGATGCGTGTGCTTTCATTTTGAGGCAAAGTAGTGAAATGTTTTCACA : 6321
AS2 : TCAGGAGCAGATTAAGTTGGAGTCTTTTCTCCTAATTGTTATGGTTGTTTGGACGGATGCGTGTGCTTTCATTTTGAGGCAAAGTAGTGAAATGTTTTCACA : 6047
      TCAGGAGCAGATTAAGTTGGAGTCTTTTCTCCTAATTGTTATGGTTGTTTGGACGGATGCGTGTGCTTTCATTTTGAGGCAAAGTAGTGAAATGTTTTCACA

             *       6340         *       6360         *       6380         *       6400         *       6420
AS1 : TTAGCAAGAAAAGAATTGACTGCTTTTGAGTCGACCTTACATGCTGGACTCCCATTCACACACAGCACGAAATAAGTCAGTCTTTACAAATGGTATTTTGAT : 6423
AS2 : TTAGCAAGAAAAGAATTGACTGCTTTTGAGTCGACCTTACATGCTGGACTCCCATTCACACACAGCACGAAATAAGTCAGTCTTTACAAATGGTATTTTGAT : 6149
      TTAGCAAGAAAAGAATTGACTGCTTTTGAGTCGACCTTACATGCTGGACTCCCATTCACACACAGCACGAAATAAGTCAGTCTTTACAAATGGTATTTTGAT
```

```
         *      6440         *      6460         *      6480         *      6500         *      6520
AS1 : AGATGCTGGATTGTTATCTGTGCCATATTTGTGCCCACTCTTTTAAGAACAATGTAGCATTATGTTCGTTTGGATAAATTGTGATTTGACAACTGATTTCAA : 6525
AS2 : AGATGCTGGATTGTTATCTGTGCCATATTTGTGCCCACTCTTTTAAGAACAATGTAGCATTATGTTCGTTTGGATAAATTGTGATTTGACAACTGATTTCAA : 6251
      AGATGCTGGATTGTTATCTGTGCCATATTTGTGCCCACTCTTTTAAGAACAATGTAGCATTATGTTCGTTTGGATAAATTGTGATTTGACAACTGATTTCAA

       *      6540         *      6560         *      6580         *      6600         *      6620         *
AS1 : TAAAACATTTGCTTCACTTAGATTTGCTGGATTTCTTAGATAATAGGAAGCCTGAGTCCTGTGTTTACTGCATCCGAAAAGCAGAGAAGCTAGAACTTTCTA : 6627
AS2 : TAAAACATTTGCTTCACTTAGATTTGCTGGATTTCTTAGATAATAGGAAGCCTGAGTCCTGTGTTTACTGCATCCGAAAAGCAGAGAAGCTAGAACTTTCTA : 6353
      TAAAACATTTGCTTCACTTAGATTTGCTGGATTTCTTAGATAATAGGAAGCCTGAGTCCTGTGTTTACTGCATCCGAAAAGCAGAGAAGCTAGAACTTTCTA

            6640         *      6660         *      6680         *      6700         *      6720         *
AS1 : ATGTCACTACACAGGGTCACTGCTTTACATCCGTTTAAGTTTCAGATTAGGAGAAGTCAGGTCAAGGGCAGAGAGGCCTCCACTGAAATAAACCAGGAGCTT : 6729
AS2 : ATGTCACTACACAGGGTCACTGCTTTACATCCGTTTAAGTTTCAGATTAGGAGAAGTCAGGTCAAGGGCAGAGAGGCCTCCACTGAAATAAACCAGGAGCTT : 6455
      ATGTCACTACACAGGGTCACTGCTTTACATCCGTTTAAGTTTCAGATTAGGAGAAGTCAGGTCAAGGGCAGAGAGGCCTCCACTGAAATAAACCAGGAGCTT

          6740         *      6760         *      6780         *      6800         *      6820         *
AS1 : TTCAGATTGAAGGTACTGAAACAATGCCACCATCTGGTGGTGCTTCCTGAAAAAGTTAGTTTTCTTTGTTAATACGTGTTAATGACAAATGTCTCTAGAAAG : 6831
AS2 : TTCAGATTGAAGGTACTGAAACAATGCCACCATCTGGTGGTGCTTCCTGAAAAAGTTAGTTTTCTTTGTTAATACGTGTTAATGACAAATGTCTCTAGAAAG : 6557
      TTCAGATTGAAGGTACTGAAACAATGCCACCATCTGGTGGTGCTTCCTGAAAAAGTTAGTTTTCTTTGTTAATACGTGTTAATGACAAATGTCTCTAGAAAG

        6840         *      6860         *      6880         *      6900         *      6920         *
AS1 : ACATTTTAAGGCTTAGCAGTAAGAATGCCTTGCCTAACAGGAATCTTGGAAAAAATTCTTTAAGTGTCAAAATGATTTCTAGTTCATGCTAATATACAAAAG : 6933
AS2 : ACATTTTAAGGCTTAGCAGTAAGAATGCCTTGCCTAACAGGAATCTTGGAAAAAATTCTTTAAGTGTCAAAATGATTTCTAGTTCATGCTAATATACAAAAG : 6659
      ACATTTTAAGGCTTAGCAGTAAGAATGCCTTGCCTAACAGGAATCTTGGAAAAAATTCTTTAAGTGTCAAAATGATTTCTAGTTCATGCTAATATACAAAAG

      6940         *      6960         *      6980         *      7000         *      7020         *      70
AS1 : ATTCTGTCAGACAATTGCTACACACAAAGGACCATCCCCTATCTACTGGAGAGTTTCTACTTTTTCATGGGTAGAGAACCGGTCTGGTGTATACACTTCTTA : 7035
AS2 : ATTCTGTCAGACAATTGCTACACACAAAGGACCATCCCCTATCTACTGGAGAGTTTCTACTTTTTCATGGGTAGAGAACCGGTCTGGTGTATACACTTCTTA : 6761
      ATTCTGTCAGACAATTGCTACACACAAAGGACCATCCCCTATCTACTGGAGAGTTTCTACTTTTTCATGGGTAGAGAACCGGTCTGGTGTATACACTTCTTA

      40         *      7060         *      7080         *      7100         *      7120         *      7140
AS1 : AATTTTACGTAAACTCAGTACTCCAGTTTGAGAACTTAAATTTCAACTGCCGATTAAATTATTTTAAAGCATAACACTCAAGCAAAACTAGAATAGTCCTTT : 7137
AS2 : AATTTTACGTAAACTCAGTACTCCAGTTTGAGAACTTAAATTTCAACTGCCGATTAAATTATTTTAAAGCATAACACTCAAGCAAAACTAGAATAGTCCTTT : 6863
      AATTTTACGTAAACTCAGTACTCCAGTTTGAGAACTTAAATTTCAACTGCCGATTAAATTATTTTAAAGCATAACACTCAAGCAAAACTAGAATAGTCCTTT

               *      7160         *      7180         *      7200         *      7220         *      7240
AS1 : TTTTTTTTTTTAGCCAAAAGACAGTTGGCTGTTAAAATGGAAAGTAGTTTCTCATAAACCAACAAATCTGATTACCAAAAAAAAAAAAAAATTGGCCATATTA : 7239
AS2 : TTTTTTTTTTTAGCCAAAAGACAGTTGGCTGTTAAAATGGAAAGTAGTTTCTCATAAACCAACAAATCTGATTACCAAAAAAAAAAAAAAATTGGCCATATTA : 6965
      TTTTTTTTTTTAGCCAAAAGACAGTTGGCTGTTAAAATGGAAAGTAGTTTCTCATAAACCAACAAATCTGATTACCAAAAAAAAAAAAAAATTGGCCATATTA

             *      7260         *      7280         *      7300         *      7320         *      7340
AS1 : GAAATAACCCCTCATGCTAAGAGCATATGGCCTTGCAGTTGGAGAACATTTAATATCCTTTTAGGAGTTATTTGCACATTTAATAGCCACTTGTGCTTAATT : 7341
AS2 : GAAATAACCCCTCATGCTAAGAGCATATGGCCTTGCAGTTGGAGAACATTTAATATCCTTTTAGGAGTTATTTGCACATTTAATAGCCACTTGTGCTTAATT : 7067
      GAAATAACCCCTCATGCTAAGAGCATATGGCCTTGCAGTTGGAGAACATTTAATATCCTTTTAGGAGTTATTTGCACATTTAATAGCCACTTGTGCTTAATT

           *      7360         *      7380         *      7400         *      7420         *      7440
AS1 : ACTAAACTTTAAAATCTCCCAATTGAAAAACACCCTCTGATCTGAAGATCTTTCTTTTGCCAGCTTGAGACTTAAAAAAAAAAAACCTAAACACTGGTGTCAA : 7443
AS2 : ACTAAACTTTAAAATCTCCCAATTGAAAAACACCCTCTGATCTGAAGATCTTTCTTTTGCCAGCTTGAGACTTAAAAAAAAAAAACCTAAACACTGGTGTCAA : 7169
      ACTAAACTTTAAAATCTCCCAATTGAAAAACACCCTCTGATCTGAAGATCTTTCTTTTGCCAGCTTGAGACTTAAAAAAAAAAAACCTAAACACTGGTGTCAA

         *      7460         *      7480         *      7500         *      7520         *      7540
AS1 : TTTCTGAAGATAACTGAAATTTGGAGATGACAGCAGTTTTTAAGGAGTTCATTTGACTTCTAATTATTGACTGACTTCCTTTCTATGGCAGGAAGAAGGTTT : 7545
AS2 : TTTCTGAAGATAACTGAAATTTGGAGATGACAGCAGTTTTTAAGGAGTTCATTTGACTTCTAATTATTGACTGACTTCCTTTCTATGGCAGGAAGAAGGTTT : 7271
      TTTCTGAAGATAACTGAAATTTGGAGATGACAGCAGTTTTTAAGGAGTTCATTTGACTTCTAATTATTGACTGACTTCCTTTCTATGGCAGGAAGAAGGTTT

       *      7560         *      7580         *      7600         *      7620         *      7640         *
AS1 : GTGCTGTTTTGAACAGATTAAAGATTTGTGTAGTTTATGACGATCATTTTGGGGGGTTTCAATTCCATCCTGCCACTCATCCTACTGGCATTTGGTCTTGGC : 7647
AS2 : GTGCTGTTTTGAACAGATTAAAGATTTGTGTAGTTTATGACGATCATTTTGGGGGGTTTCAATTCCATCCTGCCACTCATCCTACTGGCATTTGGTCTTGGC : 7373
      GTGCTGTTTTGAACAGATTAAAGATTTGTGTAGTTTATGACGATCATTTTGGGGGGTTTCAATTCCATCCTGCCACTCATCCTACTGGCATTTGGTCTTGGC

            7660         *      7680         *      7700         *      7720         *      7740         *
AS1 : CAGGTCATTCTAGCCATCACTGTTGGGTTTTCAGTGGCAGTTGCTTGGTGACTGTGGTCTTTTGATGTGCCCTGGGTTACTCCTGTTCAAGTTCTGTACCTG : 7749
AS2 : CAGGTCATTCTAGCCATCACTGTTGGGTTTTCAGTGGCAGTTGCTTGGTGACTGTGGTCTTTTGATGTGCCCTGGGTTACTCCTGTTCAAGTTCTGTACCTG : 7475
      CAGGTCATTCTAGCCATCACTGTTGGGTTTTCAGTGGCAGTTGCTTGGTGACTGTGGTCTTTTGATGTGCCCTGGGTTACTCCTGTTCAAGTTCTGTACCTG

          7760         *      7780         *      7800         *      7820         *      7840         *
AS1 : TGTCCGTTAAGCATCATTGTTTTATTAACAGGAGGAACGCTATTAACAGTAGCATGACATACAATTCTGTATTATAATTTTAAAGCTGCTGTTAAGTTGTGA : 7851
AS2 : TGTCCGTTAAGCATCATTGTTTTATTAACAGGAGGAACGCTATTAACAGTAGCATGACATACAATTCTGTATTATAATTTTAAAGCTGCTGTTAAGTTGTGA : 7577
      TGTCCGTTAAGCATCATTGTTTTATTAACAGGAGGAACGCTATTAACAGTAGCATGACATACAATTCTGTATTATAATTTTAAAGCTGCTGTTAAGTTGTGA

        7860         *      7880         *      7900         *      7920         *      7940         *
AS1 : AGTCCTTAAGCATCAAATGCAAACTGCTGAAGTTGGAGCAAGTGCCTGAACTTTGCTAATTTTTGCATTACTATGGAGCCATGTACAATAGACAGGAATGCA : 7953
AS2 : AGTCCTTAAGCATCAAATGCAAACTGCTGAAGTTGGAGCAAGTGCCTGAACTTTGCTAATTTTTGCATTACTATGGAGCCATGTACAATAGACAGGAATGCA : 7679
      AGTCCTTAAGCATCAAATGCAAACTGCTGAAGTTGGAGCAAGTGCCTGAACTTTGCTAATTTTTGCATTACTATGGAGCCATGTACAATAGACAGGAATGCA

      7960         *      7980         *      8000         *      8020         *      8040         *      80
AS1 : AGACTTGTACACTCTTGCCATTTCTTATACTTGAGGAATATAAAATGTTCCCCCTCAGGTTCTTTTGCTAATGGTACACCCTAGTTGCCTCTCTCTGCCACA : 8055
AS2 : AGACTTGTACACTCTTGCCATTTCTTATACTTGAGGAATATAAAATGTTCCCCCTCAGGTTCTTTTGCTAATGGTACACCCTAGTTGCCTCTCTCTGCCACA : 7781
      AGACTTGTACACTCTTGCCATTTCTTATACTTGAGGAATATAAAATGTTCCCCCTCAGGTTCTTTTGCTAATGGTACACCCTAGTTGCCTCTCTCTGCCACA

      60         *      8080         *      8100         *      8120         *      8140         *      8160
AS1 : CAGATAACTTTGTTCCGATATTTTCCTTTGAGATATTGAAGGGCTGGAAATGTTAGCTTTCAGTGTAAGCTTCAAGCTTAGCAGTTTTCCTTTAATCTGAGA : 8157
AS2 : CAGATAACTTTGTTCCGATATTTTCCTTTGAGATATTGAAGGGCTGGAAATGTTAGCTTTCAGTGTAAGCTTCAAGCTTAGCAGTTTTCCTTTAATCTGAGA : 7883
      CAGATAACTTTGTTCCGATATTTTCCTTTGAGATATTGAAGGGCTGGAAATGTTAGCTTTCAGTGTAAGCTTCAAGCTTAGCAGTTTTCCTTTAATCTGAGA

               *      8180         *      8200         *      8220         *      8240
AS1 : CAATATTTGATTCTTACTTCTGTTTGGGGGGGGGGGATGCAGATTTTTCATTTATCTAAATAAAATACAACGTAATTAAATACCATGGA : 8246
AS2 : CAATATTTGATTCTTACTTCTGTTTGGGGGGGGGGGATGCAGATTTTTCATTTATCTAAATAAAATACAACGTAATTAAATACCATGGA : 7972
      CAATATTTGATTCTTACTTCTGTTTGGGGGGGGGGGATGCAGATTTTTCATTTATCTAAATAAAATACAACGTAATTAAATACCATGGA
```
